# Polymerized human cord hemoglobin assisted with ascorbic acid as a red blood cell substitute alleviating oxidative stress for blood transfusion

**DOI:** 10.3389/fbioe.2023.1151975

**Published:** 2023-02-23

**Authors:** Weichen Kong, Wentao Zhou, Zeng He, Xuejun Zhang, Shen Li, Rui Zhong, Jiaxin Liu

**Affiliations:** ^1^ Institute of Blood Transfusion, Chinese Academy of Medical Sciences and Peking Union Medical College, Chengdu, Sichuan, China; ^2^ Hospital of Chengdu Office of People’s Government of Tibetan Autonomous Region, Chengdu, Sichuan, China

**Keywords:** PolyCHb, ascorbic acid, oxidative stress, kidney, Guinea pigs

## Abstract

**Introduction:** Blood transfusion is widely used in clinical settings, where considerable efforts have been devoted to develop red blood cell substitutes to overcome blood shortage and safety concerns. Among the several kinds of artificial oxygen carriers, hemoglobin-based oxygen carriers are promising due to their inherent good oxygen-binding and -loading properties. However, difficulties in prone to oxidation, production of oxidative stress, and injury in organs limited their clinical utility. In this work, we report a red blood cell substitute composed of polymerized human cord hemoglobin (PolyCHb) assisted with ascorbic acid (AA) that alleviates oxidative stress for blood transfusion.

**Methods:** In this study, the in vitro impacts of AA on the PolyCHb were evaluated by testing the circular dichroism, methemoglobin (MetHb) contents and oxygen binding affinity before and after the addition of AA. In the *in vivo* study, guinea pigs were subjected to a 50% exchange transfusion with PolyCHb and AA co-administration, followed by the collection of blood, urine, and kidney samples. The hemoglobin contents of the urine samples were analyzed, and histopathologic changes, lipid peroxidation, DNA peroxidation, and heme catabolic markers in the kidneys were evaluated.

**Results:** After treating with AA, there was no effect on the secondary structure and oxygen binding affinity of the PolyCHb, while the MetHb content was kept at 55%, which was much lower than that without AA treating. Moreover, the reduction of PolyCHbFe3+ was significantly promoted, and the content of MetHb could be reduced from 100% to 51% within 3 h. *In vivo* study results showed that PolyCHb assisted with AA inhibited the formation of hemoglobinuria, upgraded the total antioxidant capacity and downgraded the superoxide dismutase activity of kidney tissue, and lowered the expression of biomarkers for oxidative stress, e.g., malondialdehyde (ET vs ET+AA: 4.03±0.26 μmol/mg vs 1.83±0.16 μmol/mg), 4-hydroxy-2-nonenal (ET vs ET+AA: 0.98±0.07 vs 0.57±0.04), 8-hydroxy 2 deoxyguanosine(ET vs ET+AA: 14.81±1.58 ng/ml vs 10.91±1.36 ng/ml), heme oxygenase 1 (ET vs ET+AA: 1.51±0.08 vs 1.18±0.05) and ferritin (ET vs ET+AA: 1.75±0.09 vs 1.32±0.04). The kidney histopathology results also demonstrated that kidney tissue damage was effectively alleviated.

**Conclusion:** In conclusion, these comprehensive results provide evidence for the potential role of AA in controlling oxidative stress and organ injury in the kidneys induced by PolyCHb, and suggest that PolyCHb assisted with AA has promising application for blood transfusion.

## 1 Introduction

Blood transfusion is widely used in clinical settings such as trauma, severe anemia, and selective operation with massive blood loss. Among the blood components from healthy donors, the packed red blood cell is essential for maintaining the oxygen transportation function. Due to concerns about blood-borne diseases and the availability of fresh red blood cells, considerable efforts have been devoted to develop red blood cell substitutes to overcome blood shortage and safety concerns (Pape and Habler, 2007). In this regard, several artificial oxygen carriers have been reported (Khan et al., 2020). Among them, hemoglobin-based oxygen carriers (HBOCs) derived from native or re-combinational hemoglobin (Hb) through chemical modification (Alayash, 2014) are one of the most attractive category. Because of their inherent good oxygen binding and loading properties, they have become the mainstream of this field. To date, several kinds of HBOCs have undergone preclinical studies and clinical trials, suggesting that they are promising oxygen-bridging agents in many conditions (Buehler et al., 2010). However, difficulties in prone to oxidation, production of oxidative stress, and injury in organs limited their clinical utility. Thus, some strategies have been proposed, such as controlling oxidative stress by adding heme scavengers or antioxidants to the administration of HBOC.

Ascorbic acid (AA) is a first-line clinical antioxidant, widely used in a variety of situations. For example, AA has been successfully used in sickle cell disease patients and toxin-induced met-hemoglobin patients who are known to have a large amount of free ferrous and ferric Hb in circulation (Silva et al., 2013; Dhibar et al., 2018). Likewise, to alleviate the oxidative stress side effect of HBOC (Alayash, 2019), AA treatment has gained attraction to maintain infused HBOC in the reduced oxidative form (Dunne et al., 2006; Cooper et al., 2008). However, previous studies usually used rats as the animal models to evaluate their efficacy, which probably made it confused as rats bear a different antioxidant status due to their quick synthesis of endogenous ascorbic acid (Nandi et al., 1997). Among all the mammalian species, only some species, including humans, non-human primates, fruit bats, and guinea pigs are not able to synthesize endogenous AA due to an evolutionary loss of the hepatic L-gluconolactone oxidase gene (Chatterjee, 1973). The loss of endogenous AA production and subsequent the reliance on dietary intake for AA have shifted the anti-oxidative balance from plasma toward the tissue in these species (Buehler and Alayash, 2010). Therefore, it is important to consider the antioxidant status differences between different species and its impact on the study of the efficacy and safety of HBOC.

Herein, polymerized human cord hemoglobin (PolyCHb) as one kind of HBOCs was prepared by chemical cross-linking, and then *in vitro* and *in vivo* studies were performed, in which guinea pig was used as animal model to mimic the condition of human being that lacks endogenous AA production. In addition, although HBOC-induced oxidative stress can be found in several organs, such as the liver, kidney, heart, and lung (Buehler and D'Agnillo, 2010) etc., the primary safety problem of acellular Hb is the kidney, which is primarily responsible for excreting smaller degradation fragments of infused HBOC (Keipert et al., 1992). Thus, oxidative stress was mainly assayed in the kidney. In specific, guinea pigs were subjected to a 50% exchange transfusion (ET) with PolyCHb(ET group), and AA was co-administrated with PolyCHb at the end of ET (ET + AA group). By evaluating the formation of hemoglobinuria, kidney tissue oxidation stress, and pathological injury, the effects of PolyCHb assisted with AA on oxidative stress were investigated.

## 2 Materials and methods

### 2.1 Chemicals, antibodies, and kits

Human serum albumin solution was purchased from Baxter (Vienna, Austria). Vitamin C (AA) injection was supplied by Shenya Animal Healthcare (Shanghai, China). Mouse monoclonal antibodies to heme oxygenase 1 (HO-1, #MA1-112) and mouse monoclonal antibodies to L-ferritin (#MA5-14733) were purchased from Thermo Fisher Scientific (Rockford, United States). Rabbit monoclonal antibody to *ß*-Actin (#AC026) was purchased from Abclonal (Wuhan, China). Rabbit monoclonal antibody to nuclear factor erythroid 2-related factor 2 (Nrf2, # ab92946), rabbit monoclonal antibodies 4-hydroxy-2-nonenal (4-HNE, #ab46545), goat anti-mouse IgG H&L (HRP) (#ab6789), goat anti-rabbit IgG H&L (HRP) (#ab6721), a catalase (CAT) activity assay kit (#ab83464), a glutathione peroxidase (GPx) assay kit (#ab102530), and an 8-hydroxy 2 deoxyguanosine (8-OHdG) assay kit (#ab201734) were purchased from Abcam (Cambridge, MA, United States). A total antioxidant capacity (T-AOC) assay kit (#A015-2-1) and superoxide dismutase (SOD) kit (#A001-3-1) were obtained from Jiancheng Biotech. (Nanjing, China). A malondialdehyde (MDA) kit (#S0131S) was supplied by Beyotime Biotechnology (Shanghai, China). An enhanced chemiluminescence (ECL) kit (#PF001) was a product from Affinity Biosciences (Changzhou, China).

### 2.2 Preparation of PolyCHb

PolyCHb used in this study was prepared as reported previously (Zhou et al., 2015). Generally, red blood cells were separated from human cord blood (donated by Sichuan Neo-life Stem Cell Biotech INC., Sichuan, China), then stroma-free hemoglobin was derived from the red blood cells by hypnotic hemolysis. The prepared Hb was polymerized with glutaraldehyde, followed by filtration to the membrane with a molecular weight cut off at 100 kDa. The prepared PolyPHb solution was added into phosphate buffer solution (PBS) to a final concentration of 8.0 gHb/L, and bubbled with nitrogen for 10 min for oxygenation. The study protocol was approved by the Institute of Blood Transfusion Ethics Committee (Registration number:2016018).

### 2.3 CD analysis

The CD of Circular Dichroism analyzer (J-815, JASCO, Japan) was used to monitor the changes of PolyCHb secondary structure before and after AA addition. Add 30 μL PolyCHb sample solution to 3 mL 0.05 mol/L PBS solution with pH 7.5 in the cuvette for CD analysis, and then obtain CD spectrum (190–250 nm).

### 2.4 Oxygen binding affinity assay

The oxygen binding affinity of PolyCHb sample solutions before and after AA addition were detected by HEMOX-ANALYZER (TCS Scientific Corp.). The oxygen balance curve was measured at 37°C in PBS buffer solution with pH 7.4. The oxygen balance curve was used to obtain the value of P50, which was defined as the oxygen partial pressure related to the half ratio of oxygen saturated hemoglobin, and was considered as a sign of the oxygen affinity of PolyCHb samples.

### 2.5 *In vitro* antioxidant assay

PolyCHb solution (with100 μmol/L Hb concentration) containing different AA contents were prepared in 0.2 mol/L phosphate buffer saline with 100 μmol/L H_2_O_2_ (pH 7.4 PBS). By changing the AA addition amounts, the mole ratios in PolyCHb solutions between AA and PolyCHb were adjusted to be 0, 1, 2, and 3, respectively. The antioxidant reaction was started with the AA addition at the temperature of 37°C. The MetHb contents in PolyCHb solutions were tested every half an hour until 4 h.

### 2.6 Reduction of PolyCHbFe^3+^ assay

Preparation of PolyCHbFe^3+^: Dilute PolyCHb sample 40 times with PBS, add 0.1 M potassium ferricyanide solution with 1.5 times molar concentration of heme, and detect every 10 min until PolyCHb has been completely oxidized, and the content of PolyCHbFe^3+^ will not increase. Then use ultrafiltration centrifuge tube to repeatedly ultrafiltration for 6 times, and filter by 0.22 μM membrane, and calculate the prepared PolyCHbFe^3+^ heme concentration again.

By changing the AA addition amounts, the mole ratios in PolyCHbFe^3+^ solutions between AA and PolyCHbFe^3+^ were adjusted to be 0, 1, 2, and 3, respectively. The reduction reaction was started with the AA addition at the temperature of 37°C. The MetHb contents in PolyCHbFe^3+^ solutions were tested every hour until 12 h.

### 2.7 MetHb content measurement

The oxidation degrees of PolyCHb sample solutions were illustrated by the MetHb content, which was measured by the published equations (Benesch et al., 1973). Calculate the hemoglobin oxidation rate constant (Kox) according to the following formula:

$$Y=Y_{\max}\left(1-e^{-kt}\right)+Y_{0}$$

Where, t represents the measurement time point, Y represents the sample’s methemoglobin content, Y_max_ represents the highest methemoglobin content during the experiment, Y_0_ represents the initial value of methemoglobin, and K represents the sample’s oxidation rate. For oxidation reaction, the calculated K value is expressed in Koxidation, which is called K_ox_ for short. For the reduction reaction, the calculated K value is negative, so the reduction reaction can be understood as a “negative oxidation” reaction, and its absolute value is taken to represent the reduction rate Kreduction, or K_re_ for short.

### 2.8 Animals and surgical procedures

Male Hartley guinea pigs weighing 300–400 g were purchased from Chengdu Dossy experimental animals Co., Ltd. (Chengdu, China). Each guinea pig was assigned to the sham group (*n* = 5), ET group (*n* = 5), or ET + AA treatment group (*n* = 5). All animals were kept in standard conditions for 1 week as the acclimation period and they had free access to food and water. Then, the animals were restricted to food for 12 h and restricted to water for 2 h and anesthetized by IP injection of 40 mg/kg sodium pentobarbital. After shaving, a midline incision was made at the anterior neck, followed by blunt dissection to expose the right common carotid artery and the left external jugular vein. A tunnel under the skin was built using an anesthetic puncture trocar from the back of the neck to the edge of the midline incision. A PE50 tube filled with saline containing 500 IU/mL heparin was nested in the trocar and embedded in the tunnel by extracting the trocar. The outer end of the tube was connected to the heparin cap, and the inner end of the tube was intubated to each vessel. Immediately after the surgery, the animals were administrated a subcutaneous dose of meloxicam (1 mg/kg) and then were individually housed to recover for 24 h. The animal study protocol was approved by the Institutional Ethics Committee of the Institute of Blood Transfusion, Chinese Academy of Medical Sciences, and Peking Union Medical College (protocol code 2018032, approved on 31 Aug 2018).

### 2.9 Exchange transfusion operation

Fully conscious, heparinized animals underwent 50% ET with PolyCHb solution (Hb concentration 6%, human albumin 4%; the PolyCHb and human albumin were mixed immediately before ET). The blood was collected through the arterial catheter, and the PolyCHb solution was transfused through the venous catheter by syringe pumps simultaneously at a rate of 1 mL/min. The total blood volume of a guinea pig was calculated as 0.07 (milliliters per gram) × body weight (grams) (Ancill, 1956). Immediately after ET, AA injection solution was injected to the body *via* the venous catheter, by a 1 mL disposable syringe. The dosage of AA is calculated by multiplying the target concentration (2 mmol/L) with the blood volume. Blood samples (300 μL) were obtained from the arterial catheter pre-transfusion together at 0 and 4 h after ET. The guinea pigs were individually housed in metabolism cages for 4 h pre- and post-ET to collect the urine samples. The animals were sacrificed 4 h post-ET and the kidneys were dissected and rinsed with pre-cold saline. One kidney was cut in half and frozen immediately in liquid nitrogen, and the other kidney was fixed in 10% paraformaldehyde.

### 2.10 Blood/urine testing

Whole blood, collected immediately pre- and post-ET, was measured by an automated blood counter system (BC-5800, Mindray Corp., China) to record the drop in hematocrit. Urine samples were used to analyze 1) the concentration of hemoglobin (BC-5800, Mindray Corp., China) and 2) wavelength scans (DU800, Beckman) to calculate the percentage of ferric Hb, using equations for the spectrophotometric analysis of hemoglobin mixtures (Benesch et al., 1973). All urine samples (diluted five times by 0.01M, pH 7.2 PBS) and plasma samples (diluted 20 times by 0.01 M, pH 7.2 PBS) from the whole blood collected at 0- and 4-h post-ET were used to check the PolyCHb polymer distribution (HPLC platform Waters e2695 separation module and Waters e2498 UV detector). The stationary phase was Zenix SEC-300 (300 × 7.5 mm) column, the mobile phase was 0.1 M phosphate buffer, pH 6.5, the flow rate was 0.5 mL/min, and the absorbance was monitored at 410 nm.

### 2.11 Histopathology

The kidneys were fixed in 10% paraformaldehyde immediately after harvest, embedded in paraffin after dehydrating, and then 5 μm sections were cut and stained following standard hematoxylin and eosin procedures. The tissue sections were scored by a certified veterinary pathologist using semiquantitative grading criteria modified from an established one (Eadon et al., 2020). Generally, changes were recorded by the veterinary pathologist by observing the percentages of kidney parenchyma affected and then sort them to different terms (absent, mild, moderate, or diffuse). Final determinations were categorized on a scale from 0 to 3, with 0 corresponding to absence or <5% of kidney affected; 1− mild or 5%–25% of the kidney affected; 2−moderate or 26%–50% of the kidney affected; and 3−diffuse or >50% of the kidney affected.

### 2.12 Antioxidant assay

Colorimetric assay kits were used for measuring the T-AOC, CAT, SOD, and GPx activity in renal tissue homogenates. All assay procedures performed were according to the manufacturer’s instructions. The T-AOC is expressed as mmol/g protein and all the enzyme activities are expressed as U/mg protein.

### 2.13 Lipid and DNA peroxidation assay

Lipid peroxidation was assessed using a colorimetric assay kit, by measuring free MDA as decomposition products of lipid peroxides, and DNA peroxidation was measured using an enzyme-linked immunoassay kit that measures 8-OHdG as a marker of DNA peroxides. All assay procedures were performed according to the manufacturers' instructions. The values of the MDA and 8-OHdG were calculated as μmol/mg and ng/mL, respectively.

### 2.14 Western blotting

Kidney tissue (cross-sectional cut through the center of the kidney, 200 mg) was homogenized in ice-cold lysis buffer (50 μM Tris-HCl, pH 7.4, 150 μM NaCl, 0.25% deoxycholic acid, 1% NP-40, 1 mM EDTA), containing Halt Protease Inhibitors Cocktail (Thermo Fisher Scientific). Homogenates were incubated for 10 min on ice and then centrifuged at 10,000 g for 10 min. Supernatants were divided into several aliquots and stored at −80°C. The protein concentration of the aliquots was analyzed by the BCA method. The proteins were separated *via* gel electrophoresis and then transferred onto PVDF membranes. The membranes were blocked in Tris-buffered saline (pH 7.4) containing 0.01% Tween-20% and 5% skim milk. Next, they were incubated with antibodies to Nrf2, HO-1, Ferritin, and 4-HNE at 4°C overnight with gentle shaking. The proteins were visualized using the ECL kit. Equal loading was confirmed by re-probing the blots with a rabbit polyclonal antibody to *ß*-Actin. Finally, the relative intensities were calculated as a densitometric ratio between the sample and *ß*-Actin by the ImageJ program (V1.8).

### 2.15 Statistical analysis

All parameters obtained from each group were expressed as the means ± S.E.M. for the data in each group. For comparison of the urine Hb and blood hematocrit between the ET and ET + AA groups, unpaired Student’s t-tests were performed. For all the statistical comparisons among the three groups, ordinary one-way ANOVA tests with Tukey *post hoc* analysis were used. All the statistical calculations were processed by Graph Pad Prism 8.0 software (Graph Pad Software, CA, United States). A *p* < 0.05 was taken as the level of statistical significance (mark: * *p* < 0.05; ** *p* < 0.01; *** *p* < 0.001). The number of replicates in the characterization of PolyCHb is three. The number of replicates in the Western blotting was four, and the number of replicates in all other experiments in this study was five.

## 3 Results

### 3.1 CD analysis

**FIGURE 1:**
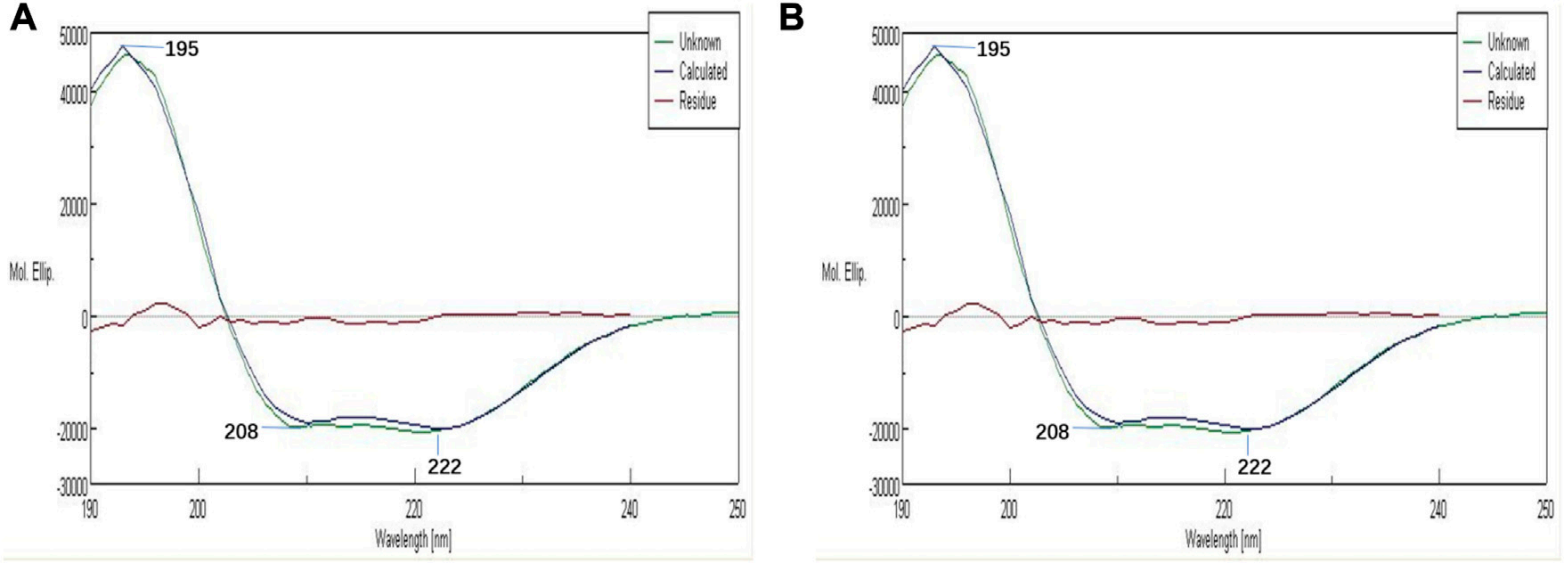
CDs of PolyCHb before **(A)** and after**(B)** AA addition.

The effects of antioxidant reaction by AA on the secondary structures of PolyCHb were investigated by the CD analysis and the results were shown in Figure 1. As reported previously, there were two negative peaks and one positive peak at the wavelength of 208, 222, and 195 nm in Figure 1A. As for the PolyCHb solution 72 h after AA addition, Figure 1B showed that the CD analysis curve presented almost no differences with that of PolyCHb solution before AA addition. Thus, it could be concluded that there was no effect of AA antioxidant reactions on the secondary structure of the PolyCHb molecules.

### 3.2 Oxygen binding affinity assay

**FIGURE 2:**
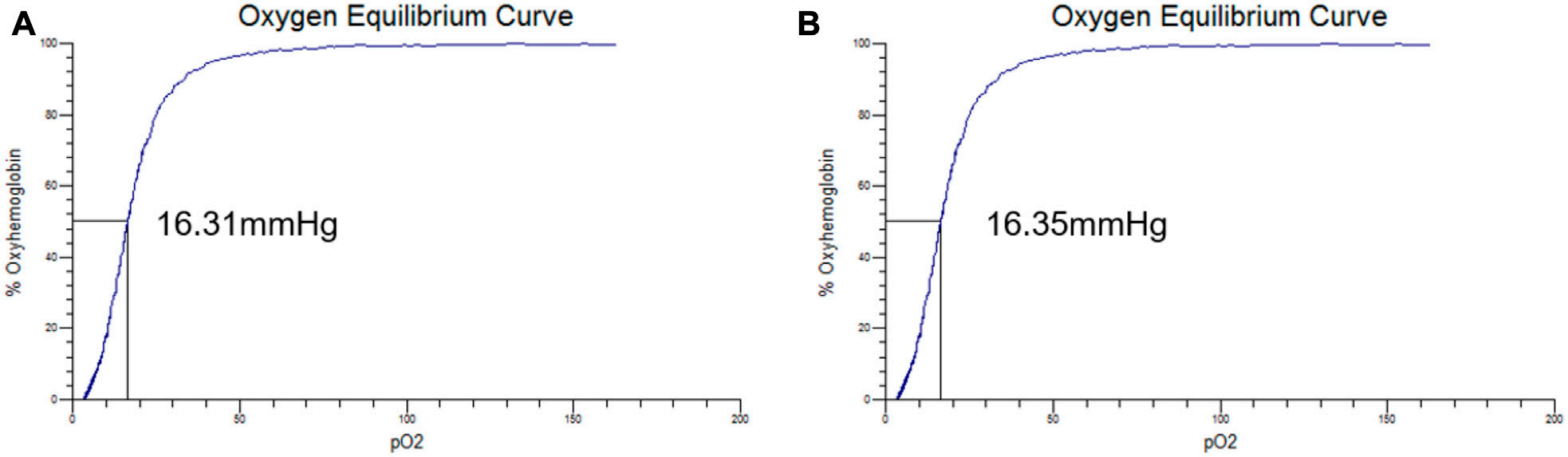
The oxygen equilibrium curve of PolyCHb, **(A)**, before AA addition, **(B)**, 72 h after AA addition.

The oxygen equilibrium curves of PolyCHb solution before and 72 h after AA addition are shown in Figures 2A, B. It could be seen that the curves before and after the AA addition were almost the same, and the P50 values presented in the two curves were about 16.31 ± 0.25 and 16.35 ± 0.31 mmHg, and Hill coefficient (Hill co) characterizing the synergistic effect between subunits of Hb solution obtained from the oxygenation curve was 2.65 ± 0.14 and 2.63 ± 0.12 (*n* = 3), which suggested that the AA addition did not alter the oxygen binding affinity of PolyCHb.

### 3.3 *In vitro* antioxidant assay

**FIGURE 3:**
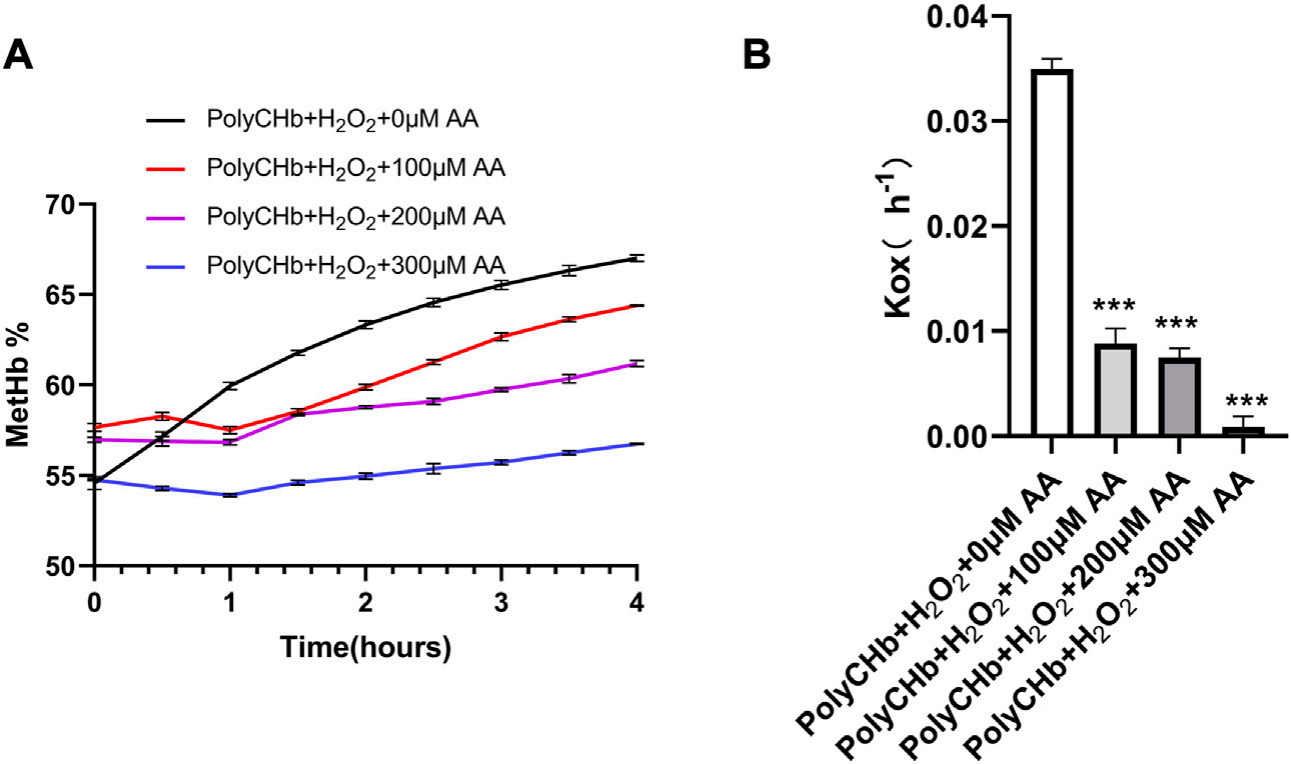
Intervention of AA on PolyCHbFe^2+^ oxidation under H_2_O_2_ at 37°C, **(A)**, the content of MetHb before and after AA addition, **(B)**, the Kox before and after AA addition, Values are expressed as mean ± SEM, *n* = 3.

As shown in Figure 3A, in the presence of H_2_O_2_ and absence of AA, the MetHb contents in PolyCHb solution increased from the initial value 49.75% to more than 65.00%, indicating the oxidant effect of H_2_O_2_ for PolyCHb. After addition of AA, the MetHb contents gradually decreased with the AA content increasing. When the AA addition amounts increased to 300 μM, the MetHb content nearly kept constantly, and the Kox was significantly lower than other groups (Figure 3B). Therefore, it could be suggested that AA plays an activity similar to catalase, reducing the oxidation of H_2_O_2_ to hemoglobin.

### 3.4 Reduction of PolyCHbFe^3+^ assay

**FIGURE 4:**
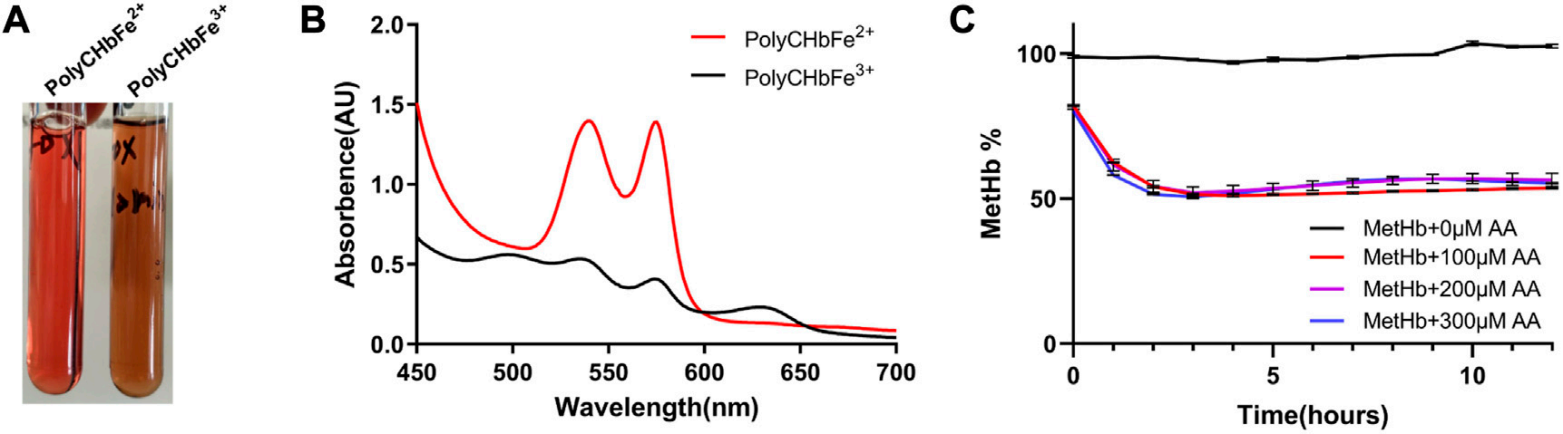
**A)**, the appearance of PolyCHbFe^2+^ and PolyCHbFe^3+^, **(B)**, the full-wavelength scanning diagram of PolyCHbFe^2+^ and PolyCHbFe^3+^, **(C)**, the content of MetHb before and after AA addition. Values are expressed as mean ± SEM, *n* = 3.

As shown in Figure 4A, the prepared PolyCHbFe^3+^ is light brown in appearance, and the content of MetHb is 99.3% ± 1.35%. Full-wavelength scanning diagram (Figure 4B) showed that PolyCHbFe^2+^ has characteristic absorption peaks at 541 and 576 nm, and the prepared PolyCHbFe^3+^ has characteristic absorption peaks at 500 and 630 nm. Compared with the group without AA, after adding 100、200、300 μM AA, the reduction of PolyCHbFe^3+^ was significantly promoted, and the content of MetHb could be reduced from nearly 100% to 51% within 3 h (Figure 4C). This suggests that AA has a strong reduction effect on the oxidized PolyCHb.

### 3.5 Ascorbic acid inhibits the formation of PolyCHb-induced hemoglobinuria

**FIGURE 5:**
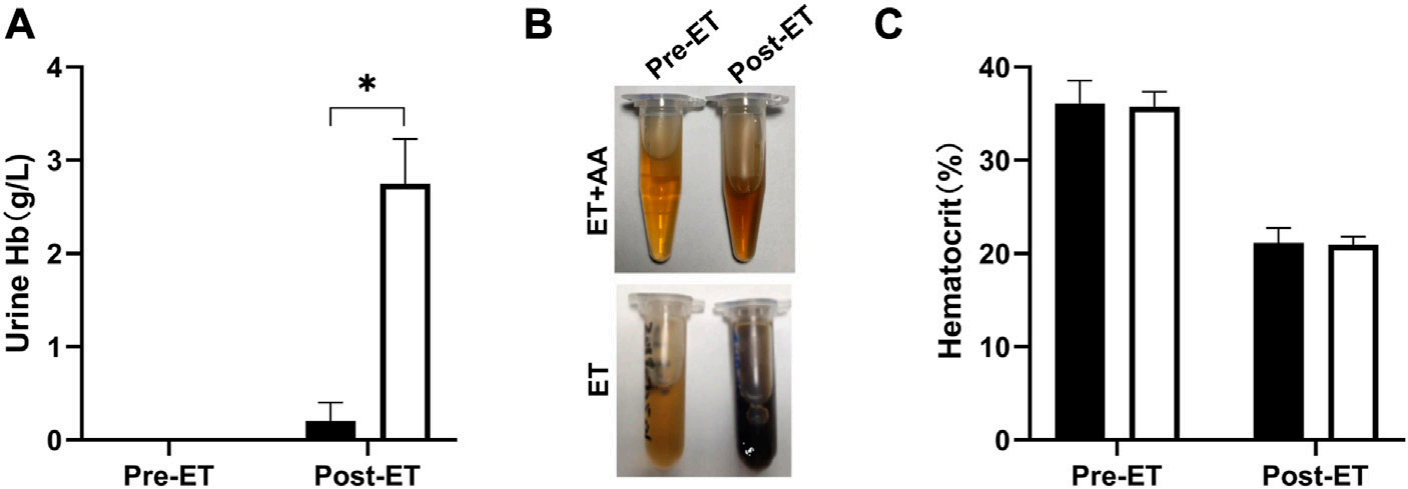
Hemoglobin levels of urine samples and hematocrit pre-ET and post-ET. **(A)** Urine Hb concentration of one animal in the ET + AA group (filled open bars) and ET group (open bars). **(B)** Picture of urine samples in the two groups. The upper picture is from one animal in the ET + AA group, the lower picture is from the ET group. In each picture, the left tube contains urine pre-ET, the right tube contains urine post-ET. **(C)** Hct drop after exchange transfusion in the ET + AA group. Values are expressed as mean ± SEM, *n* = 5.

**FIGURE 6:**
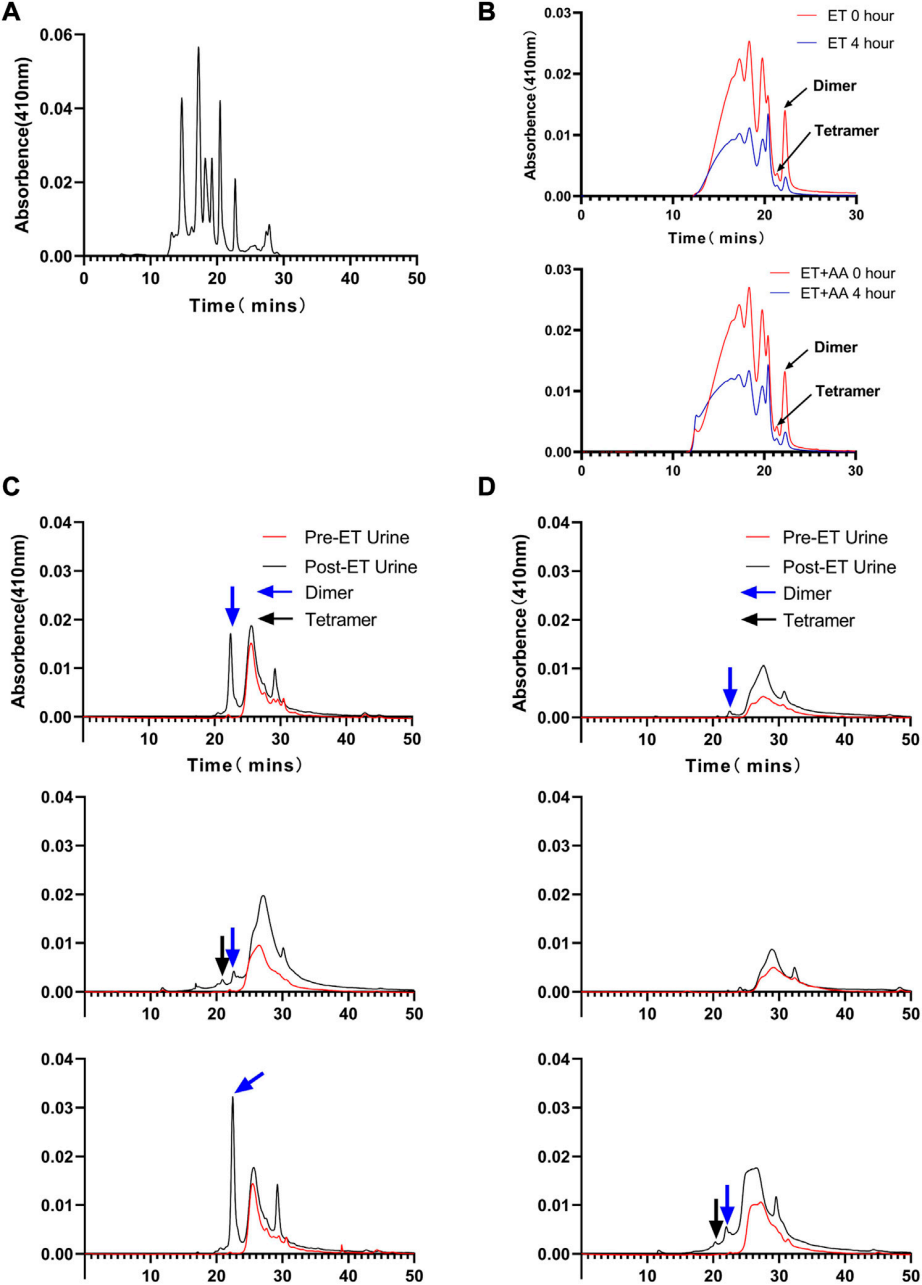
Size-exclusion chromatography distribution of heme in urine samples collected from ET group and ET + AA group. **(A)** Markers ran in parallel with samples. **(B)** Results of 0 and 4 h post-ET plasma samples; the upper panel is from one animal in the ET group, the lower panel is from one animal in the ET + AA group. **(C)** Results of three samples collected from single animals in the ET + AA group. **(D)** Results of three samples collected from single animals in the ET group.

Urine samples from individual animals were collected 4 h pre- and post-ET separately. All the urine samples collected pre-ET were clear, and no hemoglobin was detected by the blood analyzer. For the post-ET urine samples in the ET + AA group, one urine sample contained 1 g/L Hb, and the other four samples contained no Hb. Instead, Hb in urine samples in ET group increased significantly (2.75 ± 0.48 g/L). The color of all the collected urine samples was dark brown, indicating the presence of ferric hemoglobin. (Figures 5A, B); the percentage of ferric Hb was 90.20% ± 1.3%. The size-exclusion chromatography distribution curve showed that before ET, all the urine samples collected in the two groups contained trace amounts of hemoglobin in the form of dimer and degradants, although it was not detectable by the blood analyzer (Figure 5A). Compared with the markers (Figure 6A), after ET, the Hb concentration in the urine samples increased in the ET group. The major form of excreted Hb was still dimer and degradants, with a slight increase in tetramer (Figures 6C, D). It can be concluded that after the PolyCHb infusion, there was an increase in filtered hemoglobin to the kidney proximal tubular. The size-exclusion chromatography curves of infused PolyCHb in the plasma demonstrated that all the different polymer portions showed a synchronous degradation during the 4-h metabolic course (Figure 6B). The post-ET urine samples contained no significantly increased tetramer (64 kDa) or biomacromolecule with a bigger molecular weight. It can be concluded that the polymers in the infused PolyCHb were stable *in vivo*, and there was neither a quick breakdown of the polymers nor an accumulation of dimers in the circulation. The AA co-administration eliminated the phenomenon of hemoglobinuria. In addition, the ET operation was accompanied by a loss of red blood cells, which play an important role in the recycling of ascorbic acid from its oxidized forms, the ascorbate free radical and dehydroascorbic acid (May et al., 2004). Here, the drop in hematocrit was identical between the two groups (from 35.74% ± 1.61% to 20.96% ± 0.86% in the ET group vs. from 36.12% ± 2.44% to 21.14% ± 1.62% in the ET + AA group) (Figure 5C), therefore, the difference between ET group and ET + AA group has nothing to do with the loss of red blood cells during ET.

### 3.6 Histopathology

**FIGURE 7:**
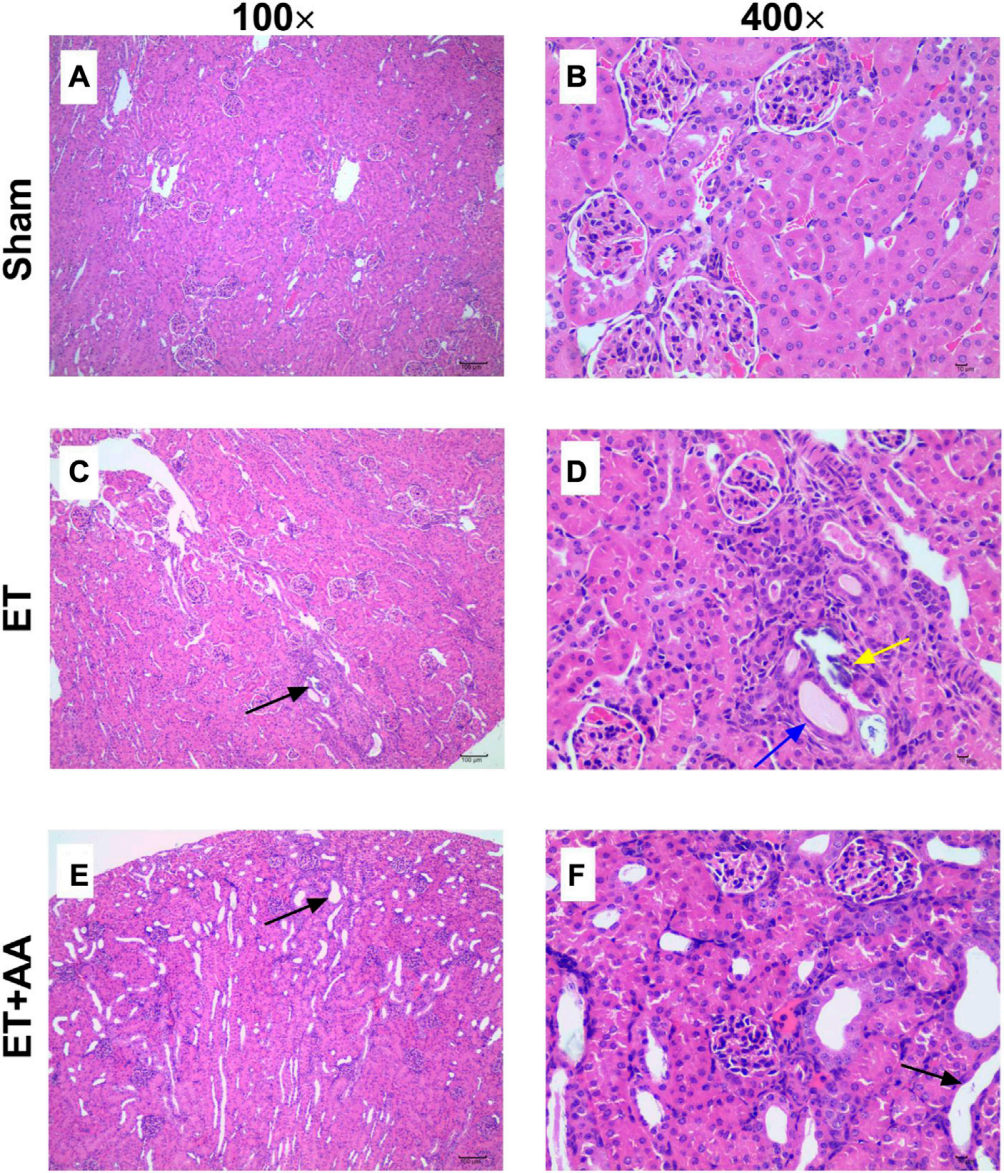
Kidney histopathological evaluation of guinea pigs in the sham, ET, and ET + AA groups. Hematoxylin and eosin-stained kidney harvested in the sham **(A,B)**, ET **(C,D)**, and ET + AA **(E,F)** groups. Magnification at ×100 **(A,C,E)** and ×400 **(B,D,F)**. In the sham group, there was no histopathological changes. In the ET group, there was local tubular epithelial necrosis [**(C)**, black arrow], glomerular micro thrombosis [**(D)**, black arrow], tubular epithelial necrosis [**(D)**, yellow arrow], and squamous epithelium [**(D)**, blue arrow]. In the ET + AA group, mild dilation of renal tubules [**(E)**, black arrow)] and flat epithelial cells [**(F)**, black arrow] can be observed.

**FIGURE 8:**
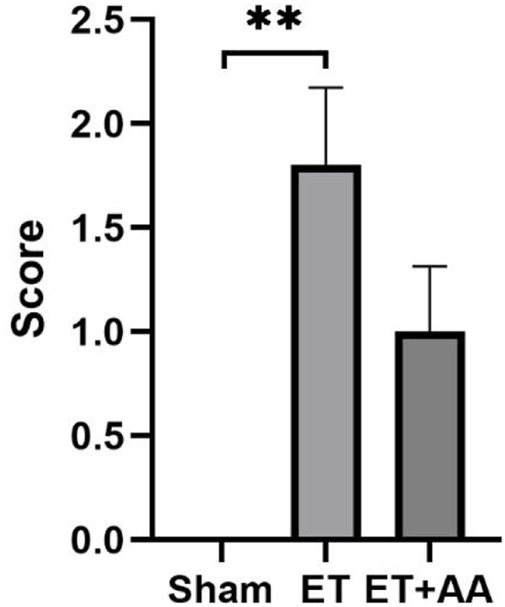
Histopathology scores of the kidneys in the sham, ET, and ET + AA groups. Values are expressed as mean ± SEM, *n* = 5.

Microscopic observation by 100 times and 400 times magnification showed the existence of histopathological change in the ET and ET + AA groups, whereas there was no microscopic change in the sham operation group (Figures 7A, B). The histopathology scores in the ET and ET + AA group were 1.8 ± 0.37 and 1.0 ± 0.32 respectively. (Figure 8).

In detail, in the sham group, the capsule of renal tissue was intact, and the boundary between cortex and medulla was clear. The glomerular structure of the cortical area was normal, and there was no obvious expansion of the capillaries and no basement membrane thickening. The renal tubular epithelial cells have normal morphology, uniform cytoplasmic staining, large and round nuclei, and no obvious degeneration and necrosis. There was no obvious fibrous tissue proliferation or inflammatory cell infiltration in the renal stroma. The structure of the medullary collecting duct was complete and clear, and no obvious pathological changes were found. However, in three cases (3/5) of ET group (Figures 7C, D), some glomerular structures in the cortex were abnormal, with capillary dilation, and a small number of lumens were filled with uniform, unstructured and slightly stained microthrombosis. In four cases (4/5), a small amount of renal tubular degeneration was found, with a slight expansion of renal tubules, flat epithelial cells, swelling of nuclei, and loss of some nuclei. One case (1/5) had local degeneration and necrosis of the renal tubular epithelium, necrosis and abscission of renal tubular epithelial cells in the necrotic area, and regeneration of renal tubular cells. Finally, In the ET + AA group (Figures 7E, F), three cases (3/5) had a small amount of local tubular degeneration, with a slight expansion of renal tubules, flat epithelial cells, swollen nuclei, and partial loss of nuclei. One case (1/5) showed local degeneration and necrosis of the renal tubular epithelium and necrosis and abscission of the renal tubular epithelial cells in the necrotic area.

### 3.7 Ascorbic acid increases the total antioxidant capacity of kidneys in exchange transfused Guinea pigs

**FIGURE 9:**
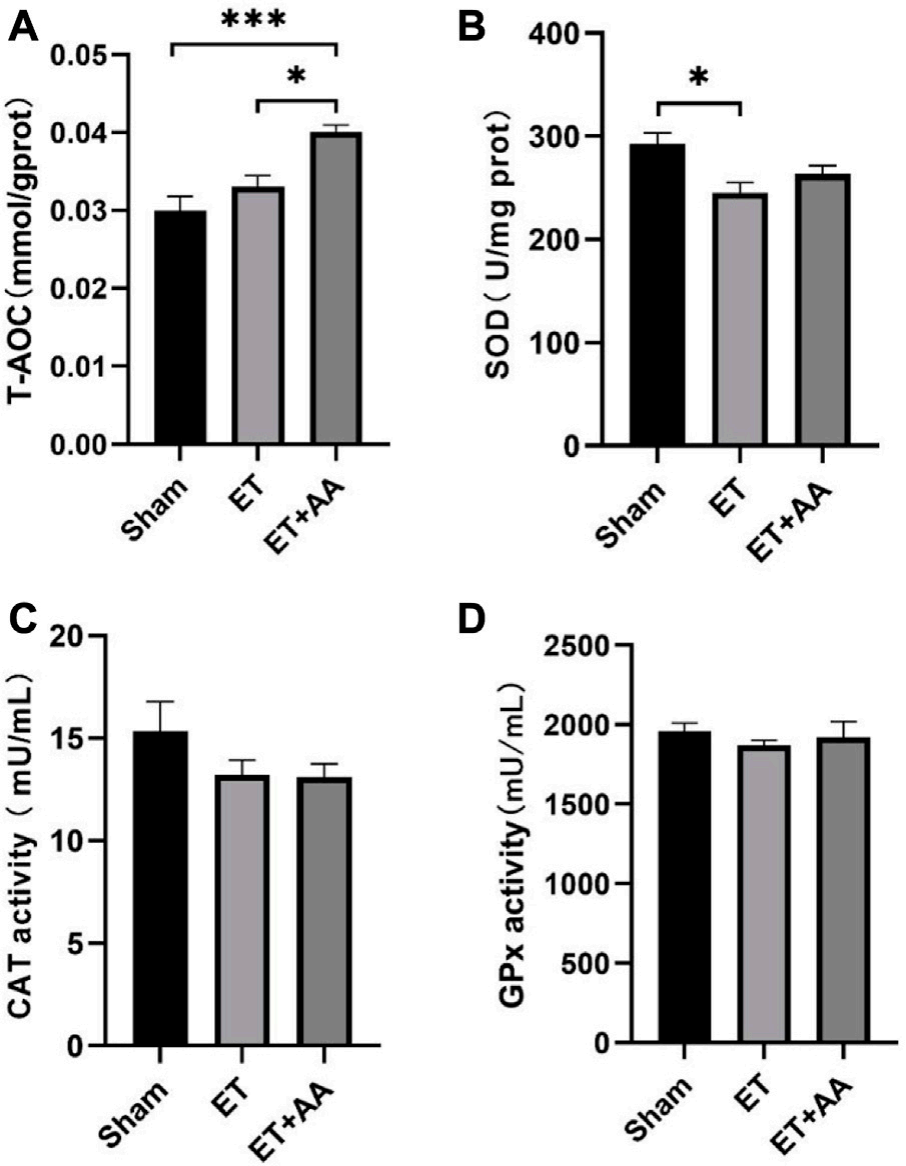
T-AOC and enzyme activities of kidney tissue 4 h post-ET in the sham, ET, and ET + AA group. **(A)** Total Antioxidant Capacity. **(B)**, SOD activity. **(C)**, CAT activity. **(D)**, GPx activity. Values are expressed as mean ± SEM, *n* = 5.

It is reported that the infusion of polymerized bovine Hb may affect the functional state of the antioxidant defense system (Rentsendorj et al., 2016), so it is speculated that PolyCHb should have a similar effect. To determine the effect of PolyCHb on the renal antioxidant enzyme response, the antioxidant enzyme activities and the T-AOC of the kidney tissue 4 h after PolyCHb exposure were measured. Compared to the sham and ET groups, the T-AOC in the ET + AA group was significantly increased (Figure 9A). SOD activity in the ET group was suppressed (Figure 9B), and CAT activity and GPx activity were not interrupted (Figures 9C, D). The detailed data are listed in the Supplementary Table S1.

### 3.8 Ascorbic acid alleviates the lipid and DNA peroxidation of the kidney

**FIGURE 10:**
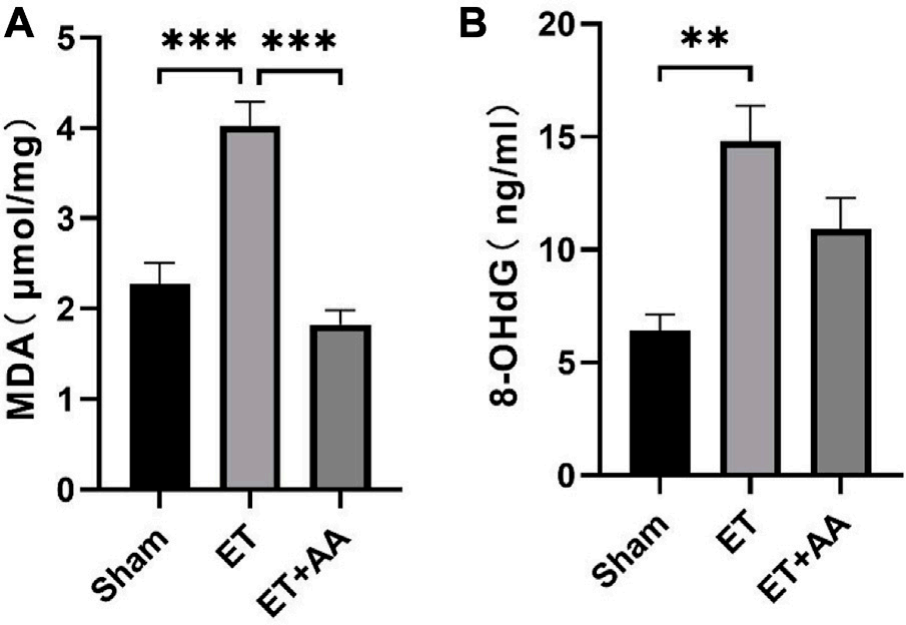
Kidney MDA and 8-OHdG levels pre- and post-ET in the sham, ET, and ET + AA groups. **(A)**, MDA levels, **(B)**, 8-OHdG levels. Values are expressed as mean ± SEM, *n* = 5.

**FIGURE 11:**
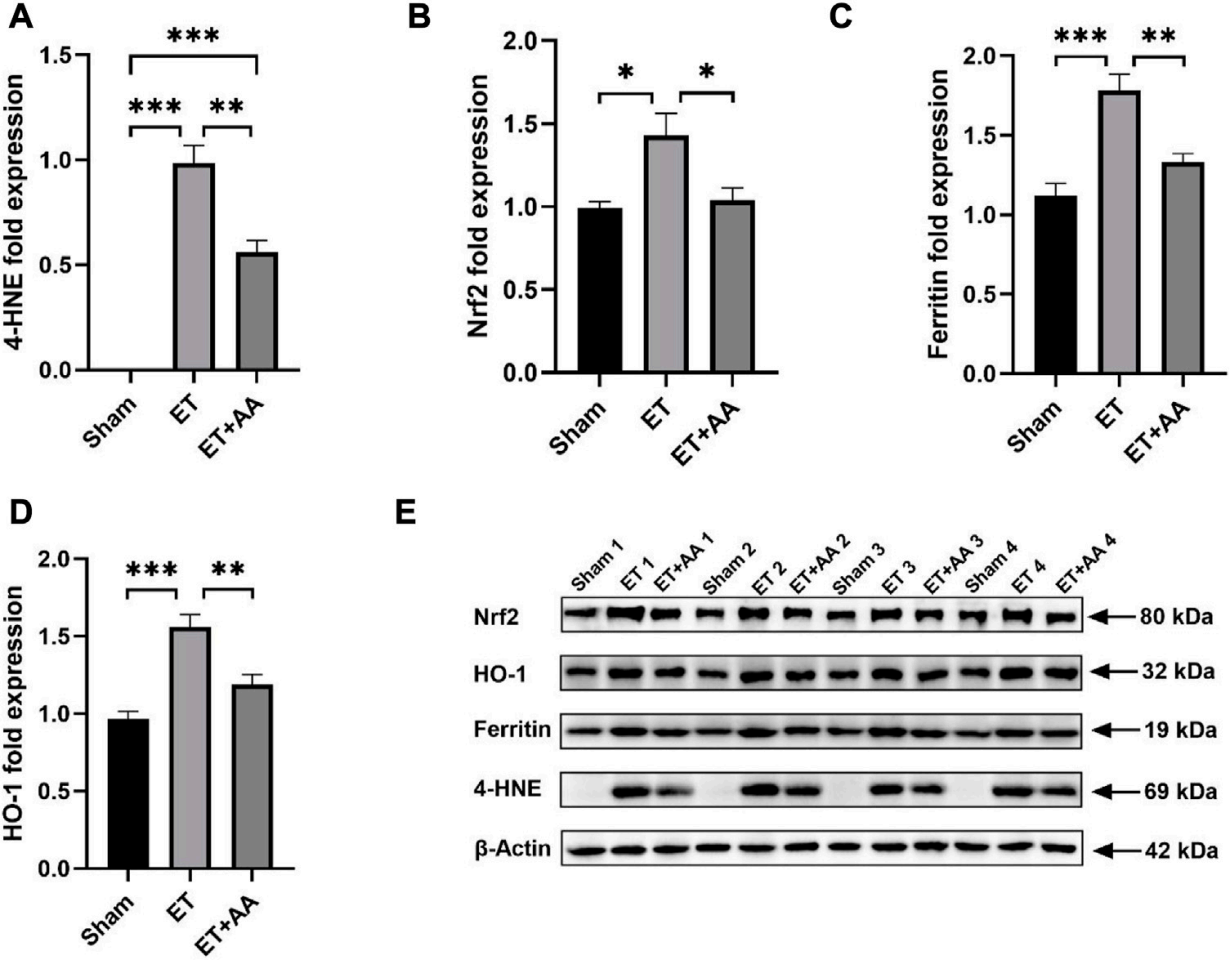
Effect of ascorbic acid on alleviating oxidative stress in the kidneys of guinea pigs. **(A–D)** Graphic representation of the fold expressions of 4-HNE, Nrf2, ferritin, and HO-1, respectively. **(E)** Image of the Western blotting assay. Values are expressed as mean ± SEM, *n* = 4

Lipid peroxidation is characterized by the oxidative degradation of polyunsaturated fatty acids attacked by free radicals. MDA is one of the commonly used biomarkers for lipid peroxidation (Frijhoff et al., 2015). In our previous research, MDA was increased in the organs of rats when they were exposed to PolyCHb. In this study, MDA increments were found in the ET group, indicating peroxidation in the kidneys of guinea pigs exposed to PolyCHb. Compared to the ET group, the MDA in the ET + AA group dropped to the base line (Figure 10A). 4-HNE is a lipid peroxidation product of n-6 polyunsaturated fatty acid, and measurement of 4-HNE-modified protein adducts has become a reliable biomarker of *in vivo* oxidative stress (Poli et al., 2008). In this study, it was found that there was no detectable 4-HNE in the sham group. However, in the ET group, there was a dramatic increase in the 4-NHE levels when they were exposed to PolyCHb. In the ET + AA group, the levels of 4-HNE were significantly lower compared to those of the ET group (Figure 11A), which indicates the effectiveness of AA on polyCHb-induced lipid peroxidation. 8-OHdG is the product of free radical-induced DNA oxidative damage and is a biomarker for oxidative stress. Similar to the results of MDA, the 8-OHdG levels increased in the kidney when PolyCHb was infused. AA can inhibit the formation of 8-OHdG when co-administrated with PolyCHb (Figure 10B). The detailed data are listed in Supplementary Table S1.

### 3.9 Ascorbic acid attenuates oxidative stress in the kidney of Guinea pigs exchange transfused with PolyCHb

It is reported that kidney exposure to Hb can activate heme catabolism pathway (i.e., HO-1, ferritin) (Butt et al., 2010) to enhance heme metabolism. The Western Blot results of 4-HNE, Ferritin and HO-1 are shown in Figure 11E, and the results were expressed by the relative expression of target protein, and the relative expression of target protein = the integral optical density (IOD) of target protein/the integral optical density (IOD) of internal reference. Figure 11B showed that Nrf2 expression increased in the ET group compared to the sham and ET + AA groups. HO-1 and ferritin expression increased in the kidney tissue in the ET group, and AA lowered their expression levels to the baseline. (Figures 11C, D). Thus, it can be deduced that in the ET group, there was a higher degree of oxidative stress, and the system upregulated the Nrf2 to prepare to further strengthen the activity of antioxidant enzymes. The detailed data are listed in the Supplementary Table S1.

## 4 Discussion

Hemoglobin functions as an oxygen supplier through the process of combining and releasing oxygen [HbFe^2+^+O_2_ ⇄HbFe^2+^(O_2_)]. In this process, hemoglobin can also be oxidized to MetHb, which generates superoxide ions. Therefore, it should pay attention to the autoxidation of hemoglobin on the HBOC research. In this study, PolyCHb was prepared from HbF by chemical crosslinking, and the results of adding AA to PolyCHb indicated that there was no effect of AA antioxidant reactions on the secondary structure of the PolyCHb molecules and the oxygen binding affinity of PolyCHb. In addition, AA plays an activity similar to catalase, reducing the oxidation of H_2_O_2_ to hemoglobin and has a strong reduction effect on the oxidized PolyCHb, which plays an important role in maintaining the status of reduced hemoglobin in the body.

*In vivo* study, AA was used as a small molecular antioxidant to evaluate the effect of hemoglobin oxidation on renal stress in guinea pig exchange model. AA co-administration with PolyCHb was compared with the sham group and the ET group in terms of renal oxidative stress. It has been proved that PolyCHb infusion causes oxidative stress and damage to the kidney of guinea pigs. Furthermore, it was also found that AA had a significant effect on controlling the oxidative stress in the kidneys and inhibiting the formation of hemoglobinuria after PolyCHb infusion.

Under normal physiology conditions, Hb is packed in the red blood cells and protected by enzymes such as CAT, GSH, and GPx to prevent oxidation (Rifkind et al., 2014). Transfusion of HBOC into the blood circulation can “create a potentially dangerous oxidative environment by generating redox oxygen radicals” (Buehler and Alayash, 2010), and actually, oxidative stress is a major safety concern for the clinical application of HBOC. Different from the control of Hb auto-oxidation in the RBC, the control of circulating HBOC in plasma is not directly enzymatic but instead controlled by small molecule reducing agents, such as AA, and GSH (Buehler and Alayash, 2010). Under such circumstances, “endogenous plasma-reducing systems may be easily overwhelmed by the infusion of a high-dose of HBOC” (D’Agnillo, 2013). Exchange transfusion in this study caused a heavy exposure of PolyCHb in the blood circulation. The PolyCHb solutions used in this study contained less than 3% dimer, and hemoglobinuria did not occur when it was transfused to rats in our previous studies. However, in this study, hemoglobinuria formed post-ET in the guinea pigs in the ET group. This difference between rats and guinea pigs confirms the necessity of using guinea pigs in the preclinical study of PolyCHb. In addition to our findings, other studies have also demonstrated that when transfused with polymerized bovine Hb, hemoglobinuria could also be found in guinea pigs (Boretti et al., 2009; Butt et al., 2010). Considering that guinea pigs are prone to form hemoglobinuria when exposed to HBOC, together with the fact that there was no hemoglobinuria in the ET + AA group, AA showed a solid effect on eliminating the formation of hemoglobinuria in this study. This effect is of great significance to the clinical application of HBOC. The reason is that if hemoglobinuria occurs during the clinical application of HBOC, it is difficult for doctors to find out the reason, because it may be due to the dimer in HBOC, or hemolysis or rhabdomyolysis. Thus, from a clinical point of view, if AA is further confirmed to inhibit the formation of hemoglobinuria in clinical trials, hemoglobinuria will not be a major concern when using PolyCHb.

Acellular hemoglobin is particularly prone to oxidation and then denaturation, accompanied by heme release and free radical formation (Reeder, 2010), especially dimers are extremely prone to oxidation (Marden et al., 1995). In this study, the urine samples of the ET group were confirmed to contain ferric Hb dimers. The dimers in urine were filtered from the circulation through glomeruli and then taken up by proximal tubular cells. Further, they were excreted when the tubular reabsorption capacity was exceeded. In the process of heme catabolism, heme oxygenase (HO) is a rate-limiting enzyme, which can be upregulated by heme and hypoxia. There are two isoforms of HO—HO-1 and HO-2. HO-1 is the inducible isoform, expressed at a low level in the kidney under basal conditions, but its expression is regulated by a wide variety of physiologic and pathophysiologic stimuli (Drummond et al., 2019), and it plays a protective role in the heme protein-mediated renal injury (Nath et al., 1995). Additionally, the nuclear factor erythroid 2-related factor 2 (Nrf2)-Kelch-like ECH-associated protein 1 (KEAP1) regulatory pathway plays a vital role in protecting cells and tissues from oxidative damage (Panieri et al., 2020). The mechanism of Nrf2 in the defense against oxidative stress is that it can activate the expression of antioxidant enzymes and HO-1 (Rubio-Navarro et al., 2019). In this study, PolyCHb infusion in the ET group was accompanied by the elevation of Nrf2. AA treatment lowered the level of Nrf2, indicating the body’s response to PolyCHb infusion by activating the anti-oxidative system. The enzyme activity had no significant alarm 4 h post-ET; we assume that this is because it takes a period of time from the upregulation of Nrf2 to the activation of downstream enzyme expression. Observation more than 4 h after ET will help to find further relationship.

As AA can eliminate the hemoglobinuria in this study, the difference of HO-1 and ferritin between the ET and ET + AA groups proved that AA upregulated the Hb reabsorption capacity of proximal tubular cells, and the filtered dimers were reabsorbed in the renal tubules. In contrast, in the ET group without AA prevention, filtered dimers were not fully reabsorbed and were pooled in the bladder. HO catalyzes the degradation of heme into biliverdin, iron, and CO, and it is an important molecular in protecting against acute heme protein-induced nephrotoxicity and other forms of acute tissue injury (Nath et al., 2001; Tracz et al., 2007). HO-1 upregulation is often accompanied by ferritin induction, aim to trap iron released from Hb subunit and limit oxidative stress (D’Agnillo, 2013). Another study demonstrated that HO-1 induction was beneficial for protecting the kidneys from injury by *in vitro* and preclinical models (Nath and Agarwal, 2020). In this study, the expressions of HO-1 and ferritin in the ET group was higher than that in sham group, indicating the presence of metabolic stress for heme in this group. AA treatment lowered the expressions of HO-1 and ferritin in the ET + AA group, and decreased other oxidative biomarkers, including MDA, 4-HNE, and 8-OHdG. Along with the histopathological changes, AA showed an exact protection role in alleviating the oxidative stress in kidneys when co-administrated with PolyCHb.

As an electron donor, AA is a potent water-soluble antioxidant. Its antioxidant effect has been demonstrated in many applications. For example, AA has been widely used for the reduction of lipid peroxidation in cancer treatment (Reang et al., 2021), oxidative damage induced by degenerative diseases, and other stimulations (Ghosh et al., 1996; Panda et al., 2000). Another beneficial characteristic of AA is the wide-ranging safety, and it is safe when even used at high doses. When using HBOC, the difference between AA-producing animals and non-AA-producing animals also indicates the important role of AA in previous studies. Additionally, this study inspired and confirmed that the selection of animal species is important during the preclinical study of PolyCHb. Usually, rats are the most commonly used animal in the standard pre-clinical toxicology evaluation of HBOC (Newmark, 2014), but guinea pigs should be added to the evaluation list to focus on the evaluation of oxidative stress and renal side effects of the acellular Hb-based blood substitute.

There are still several limitations of this study. Firstly, although we proved that AA could prevent the formation of methemoglobinuria after PolyCHb infusion, the exact mechanism should be further investigated. Secondly, the optional dosing and co-administration method of AA together with PolyCHb infusion needs to be further studied in detail. Finally, more test time points, for example, 8, 12, 24, and 48 h, should be added to confirm the changes during the dynamic time course and the best AA prevention strategy.

## Data Availability

The original contributions presented in the study are included in the article/Supplementary Material, further inquiries can be directed to the corresponding author.
