# Supplementary material for: Polymerized human cord hemoglobin assisted with ascorbic acid as a red blood cell substitute alleviating oxidative stress for blood transfusion
Source: Front Bioeng Biotechnol. 2023 Feb 23;11:1151975. doi: 10.3389/fbioe.2023.1151975 (PMC9995943; doi:10.3389/fbioe.2023.1151975)
Supplement: Supplementary file 1 [file Table1.DOCX]

Supplementary Materials

**Table. S1.** Data for T-AOC, enzyme activities, and oxidative stress marker measurements in the sham, ET, and ET+AA groups.

| **Group** | **Shame** | **ET** | **ET+AA** |
| --- | --- | --- | --- |
| T-AOC（mmol/mg） | 0.030±0.002 | 0.033±0.001 | 0.040±0.001 |
| SOD activity（mg protein/mL） | 292.99±10.45 | 245.21±9.79 | 263.51±8.17 |
| CAT activity（μmol/mg） | 15.34±1.43 | 13.21±0.74 | 13.11±0.64 |
| GPx activity （mU/mL） | 1958.22±53.94 | 1872.49±29.26 | 1922.83±96.00 |
| MDA （μmol/mg） | 2.28±0.23 | 4.03±0.26 | 1.83±0.16 |
| 8-OHdG（ng/mL） | 6.43±0.70 | 14.81±1.58 | 10.91±1.36 |
| 4-HNE fold expression | 00.00±00.00 | 0.99±0.08 | 0.56±0.06 |
| Nrf2 fold expression | 0.99±0.04 | 1.43±0.13 | 1.04±0.07 |
| Ferritin fold expression | 1.12±0.08 | 1.78±0.10 | 1.33±0.05 |
| HO-1 fold expression | 0.97±0.05 | 1.56±0.08 | 1.19±0.06 |
